# Casual C peptide index: Predicting the subsequent need for insulin therapy in outpatients with type 2 diabetes under primary care

**DOI:** 10.1111/1753-0407.13257

**Published:** 2022-03-01

**Authors:** Ryota Uehara, Eijiro Yamada, Yasuyo Nakajima, Aya Osaki, Shuichi Okada, Masanobu Yamada

**Affiliations:** ^1^ Department of Medicine and Molecular Science Gunma University Graduate School of Medicine Maebashi Japan

**Keywords:** casual examination, C‐peptide index, type 2 diabetes, urinary C‐peptide/creatine ratio, 随机检查, C肽指数, 2型糖尿病, 尿C肽/肌酸比值

## Abstract

**Background:**

Evaluation of residual beta cell function is indispensable in patients with type 2 diabetes as it informs not only diagnoses but also appropriate treatment modalities. However, there is a lack of convenient biomarkers for residual beta cell function. Therefore, we evaluated endogenous insulin level as a biomarker in outpatients who were being treated with insulin therapy and in patients who were introduced to insulin therapy after 4 years.

**Methods:**

Data of 174 outpatients with type 2 diabetes (50% male) whose glycemia was moderately controlled (glycated A1c 7.3% [5.2%–14.8%]) were reviewed. Twenty patients whose estimated glomerular filtration rate was lower than 30 ml/min/1.73 m^2^ were excluded from the evaluation of endogenous insulin level with both casual C‐peptide index (C‐CPI) and urinary C‐peptide/creatinine ratio (determined at any time, generally 1–2 h after breakfast). Patients were stratified based on the provision of insulin therapy.

**Results:**

C‐CPI and UCPCR were significantly lower in the insulin‐treated patients than in the insulin‐untreated patients (0.9 vs. 2.2, *p* < 0.0001; 24.7 vs. 75.5, *p* = 0.0003, respectively). Moreover, C‐CPI were significantly lower in the insulin‐requiring patients for 4 years than in the insulin‐unrequiring patients (1.0 vs. 1.7, *p* = 0.0184). The multivariate logistic regression analysis revealed that both indicators of insulin secretion influenced the requirement for insulin therapy, but C‐CPI could serve as the most convenient and useful biomarker for not only current insulin therapy requirements (*p* = 0.0002) but also the subsequent requirement for insulin therapy (*p* = 0.0008).

**Conclusions:**

C‐CPI could be determined easily, and it was found to be a more practical marker for outpatients; therefore, our findings would have critical implications for primary care.

## Graphical abstract

**Highlights**

- Analyses of the factors affecting insulin introduction in the 4‐year period from 2016 and 2020 were conducted. Multivariate logistic regression analysis revealed that C‐CPI contributed the most. The C‐CPI may serve as a convenient useful biomarker for not only the requirement current insulin treatment but also the requirement of insulin therapy for 4 years.
- C‐CPI could be determined easily.
- It can be a convenient biomarker for predicting the requirement for insulin therapy.

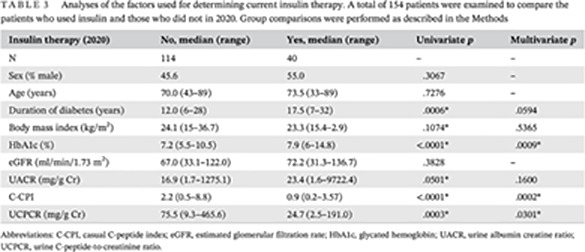

## 摘要

**背景:**

对2型糖尿病患者残余β细胞功能的评估是必不可少的, 因为它不仅指导诊断, 而且指导适当的治疗方式。然而, 尚缺乏方便评估残余β细胞功能的生物标志物。因此, 我们评估了正在接受胰岛素治疗的门诊患者和在4年后接受胰岛素治疗的患者内源性胰岛素水平。

**方法:**

回顾174例门诊2型糖尿病患者(男性占50%), 血糖得到适度控制(HbA1c:7.3%, 5.2%~14.8%)。20例肾小球滤过率低于30mL/min/1.73m^2^的患者被排除在评估内源性胰岛素水平之外, 评估根据随机C肽指数(C‐CPI)和尿C肽/肌酐比值(UCPCR, 随时测定, 一般在早餐后1~2h)。根据胰岛素治疗的情况对患者进行分层。

**结果:**

胰岛素治疗组的C‐CPI和UCPCR显著低于非胰岛素治疗组(分别为0.9比2.2, p < 0.0001, 24.7和75.5, p = 0.0003)。此外, 4年来需要胰岛素的患者的C‐CPI显著低于不需要胰岛素的患者(1.0比1.7, p= 0.0184)。多变量逻辑回归分析显示, 胰岛素分泌的两个指标都会影响胰岛素治疗的需求, 但 C‐CPI 可以作为最方便和有用的生物标志物, 不仅可以反映当前的胰岛素治疗需求(p = 0.0002), 还可以指导后续胰岛素治疗的需求(p = 0.0008)。

**结论:**

C‐CPI易于测定, 是门诊患者较为实用的指标, 因此, 我们的发现对初级保健具有重要意义。

## 1 INTRODUCTION

Type 2 diabetes is characterized by insulin resistance and deficient insulin secretion, although it does not develop only in response to insulin resistance.^1^, ^2^ Insulin resistance necessitates more insulin to maintain blood glucose; therefore, not only the number of pancreatic beta cells but also insulin production per cell must be increased to compensate for this deficit.^3^ This continued load on beta cells may lead to apoptotic cell death and the development of diabetes.^1^, ^3^, ^4^ In this respect, the residual function of pancreatic beta cells may already be approximately 50% of the normal level during diabetes development.^5^ After the development of diabetes, this load may result in a progressive decline in pancreatic beta cell function, thereby necessitating insulin therapy. Therefore, evaluation of residual insulin secretion during the course of type 2 diabetes is important when choosing between insulin therapy and other treatments, as diabetes is a lifelong condition.^1^, ^4^, ^6^ Relevant research is of clinical significance for the development of new therapies aimed at improving insulin secretion.^4^, ^7^

C‐peptide immunoreactivity (CPR) is commonly used to evaluate endogenous insulin level. Insulin is generated upon the enzymatic cleavage of its precursor, proinsulin, in pancreatic beta cells and is subsequently secreted into the blood (both insulin and CPR are secreted in equal amounts).^8^, ^9^ Although the insulin concentration can be measured using immunoassays, it is often unstable, resulting in inconsistent measurements.^10^ Moreover, insulin concentration can be measured, but exogenous and endogenous insulin cannot be distinguished in patients on insulin therapy. However, unlike insulin, the physiological function of CPR for improved glycemic control in diabetes is still controversial.^8^, ^9^ CPR is more stable than insulin; therefore, CPR level is evaluated more commonly to predict the need for insulin therapy.^11^, ^12^ It is important to evaluate the CPR level adjusted to blood glucose level to evaluate the function of residual pancreatic beta cells. In this respect, determining the CPR index (C‐peptide index [CPI]), that is, fasting serum CPR (ng/ml)/fasting blood glucose (mg/dl) × 100, is considered an appropriate approach.^9^, ^11^, ^13^ The CPI is widely used to asses endogenous insulin secretary reserves in both type 1 and type 2 diabetes. Moreover, it could be the indicator for insulin requirements for appropriate glycemic control in type 2 diabetes.^11^, ^12^, ^13^

Although the CPI can be used as a factor to evaluate the function of residual pancreatic beta cells, its normal range is determined when fasting.^11^, ^13^ Considering that most patients with type 2 diabetes are managed via primary care, it is often difficult to check the blood of all patients while they are fasting. Moreover, the CPI during fasting might not reflect the additional secretion of insulin as a result of food intake, and this is also important when evaluating the function of residual pancreatic beta cells. Therefore, measurement of the casual CPI (C‐CPI) in outpatients at any time may prove to be more efficient; however, to date, only a few studies have investigated the applicability of the C‐CPI in outpatients. Additionally, the use of the urinary C‐peptide/creatinine ratio (UCPCR) has been reported as a noninvasive and convenient new marker for insulin secretion.^14^, ^15^ However, serum CPR is restricted to the hospital setting and requires serum separation by centrifugation and subsequent freezing; in contrast, the UCPCR is stable at room temperature for up to 3 days.^15^ Importantly, the correlation between serum CPR and UCPCR has not yet been fully evaluated.

In the present study, we reassessed the characteristics of Japanese outpatients with type 2 diabetes requiring insulin therapy by measuring the C‐CPI and UCPCR to identify the requirement for insulin therapy. We also examined the characteristics of outpatients with type 2 diabetes requiring insulin therapy after 4 years, which, to the best of our knowledge, is the longest observational period in such studies.

## 2 METHODS

### 2.1 Ethics statement

This study was approved by the Gunma University Institutional Review Board and conformed to the tenets of the Declaration of Helsinki (revised in Fortaleza, Brazil; October 2013). All patients provided written informed consent before undergoing any study‐related procedures.

### 2.2 Subjects

The data of all outpatients with type 2 diabetes visiting the Division of Endocrinology and Diabetes, Keiaido Hospital, were reviewed, and patients whose CPR was measured in 2016 were selected. Patients whose estimated glomerular filtration rate (eGFR) was lower than 30 ml/min/1.73 m^2^ were excluded as the CPR in them would be unstable and inaccurate.^16^

### 2.3 Measurements

Endogenous insulin concentration was estimated by measuring the casual serum CPR level, casual blood glucose, or UCPCR evaluated at any time point during the visit (normally up to 1–2 h after breakfast). Both serum and urine CPR levels (ng/ml) were examined using the chemiluminescent immunoassay with the ARCHITECT i2000SR immunoassay analyzer (Abbot Japan) by LSI Medience Corporation, Inc. (Tokyo, Japan). The C‐CPI was calculated using the following equation: casual serum CPR (ng/ml)/casual blood glucose (mg/dl) × 100.

### 2.4 Statistical analysis

Data are presented as median (range) and percentage for frequency variables. Results are expressed as the average value for continuous variables or as value and percentage for categorical variables. Group comparisons were performed using the analysis of variance and Wilcoxon rank‐sum test for continuous variables without normal distribution. The variables found to be significant in the univariate analysis (*p* < .15) were included in the multivariate models. Associations between continuous variables were examined using Spearman's correlation coefficient analysis. All tests of significance and the resulting *p* values were two sided, and the level of significance was set at 5%. The statistical analyses were performed using JMP Pro 15.2.0 software (SAS Institute, Cary, NC, USA).

## 3 RESULTS

We screened 174 outpatients with type 2 diabetes (50% male) whose glycemia was moderately controlled (glycated A1c 7.3% [5.2%–14.8%]). The characteristics of the 174 patients are presented in Table 1. Twenty patients with the eGFR lower than 30 ml/min/1.73 m^2^ were excluded from the evaluation endogenous insulin levels with both C‐CPI and UCPCR. Profiles of all 154 enrolled patients are provided in Table 2A and B. The median age of the patients was 71.0 years, and 48.4% of the patients were male; the median duration of diabetes was 13.0 years. We evaluated the correlation of C‐CPI and UCPCR with sex and confirmed that the relationships were similar (data not shown). In 2016, the median body mass index of the patients was 23.9 kg/m^2^, which significantly decreased in 2020 (23.8 kg/m^2^, *p* = .0188). Although the glycated hemoglobin (HbA1c) level and eGFR did not significantly change from the baseline, the urine albumin creatine ratio significantly increased (11.6 vs. 17.4 mg/g Cr, *p* = .0353; Table 2B). Similarly, the C‐CPI significantly increased in 2020 compared with that in 2016 (Table 2B).

**TABLE 1:** Characteristics of all patients in this study

All subjects	
N	174
Sex (% male)	50
Age (years)	71.0 (33–93)
Duration of diabetes (years)	9.0 (2–30)
Body mass index (kg/m^2^)	23.8 (15–38)
HbA1c (%)	7.3 (5.2–14.8)
Stages of diabetic kidney disease	
No nephropathy (%)	58.6
Microalbuminuria (%)	28.7
Macroalbuminuria (%)	8
Elevated plasma creatinine (%)	4.5
Treatment	
Sulfonylurea (%)	13.2
Glinide (%)	20.7
Dipeptidyl peptidase‐4 inhibitor (%)	47.1
Biguanide (%)	40.2
α‐glucosidase inhibitor (%)	39.7
Sodium glucose cotransporter 2 inhibitor (%)	33.3
Thiazolidine (%)	0.0
Glucagon‐like peptide‐1 receptor agonist (%)	13.8
Insulin (%)	29.9

Abbreviation: HbA1c, glycated hemoglobin.

**TABLE 2:** Characteristics of patients included in this study. (A) Basic characteristics in 2020 of 154 patients included in this study. (B) Characteristics of 154 patients at 2016 and 2020 were compared. Group comparisons were performed as described in the Methods

A			
Subjects (2020)	Median (range)		
N	154		
Sex (% male)	48.4		
Age (years)	71.0 (33–89)		
Duration of diabetes (years)	13.0 (6–32)		

B			
Year	2020, median (range)	2016, median (range)	*p*
Body mass index (kg/m^2^)	23.8 (15–36.7)	23.9 (15.4–37.4)	.0188*
HbA1c (%)	7.3 (5.5–14.8)	7.1 (5.5–14.3)	.3847
eGFR (ml/min/1.73 m^2^)	68.2 (31.3–136.7)	67.9 (34.2–179.0)	.7937
UACR (mg/g Cr)	17.4 (1.6–9722.4)	11.6 (0–815.4)	.0353*
C‐CPI	1.7 (0.2–8.8)	1.3 (0.1–4.4)	*<*.0001*
UCPCR (mg/g Cr)	63.0 (2.5–465.6)	ND	ND
Sulfonylurea (%)	14.3	18.2	–
Glinide (%)	21.4	14.9	–
Dipeptidyl peptidase‐4 inhibitor (%)	50.6	55.2	–
Biguanide (%)	44.2	39.0	–
α‐glucosidase inhibitor (%)	42.9	48.1	–
Sodium glucose cotransporter 2 inhibitor (%)	33.1	17.5	–
Thiazolidine (%)	0.0	2.6	–
Glucagon‐like peptide‐1 receptor agonist (%)	13.0	9.1	–
Insulin (%)	26.0	24.7	–

Abbreviations: C‐CPI, casual C‐peptide index; eGFR, estimated glomerular filtration rate; HbA1c, glycated hemoglobin; UACR, urine albumin creatine ratio; UCPCR, urine C‐peptide‐to‐creatinine ratio.

With respect to the treatment type, whereas the usage of sulfonylurea in 2020 decreased by approximately 4% compared to that in 2016, that of glinide/biguanide and sodium glucose cotransporter 2 (SGLT2) inhibitors in 2020 increased by more than 5% compared to that in 2016. In particular, the use of SGLT2 inhibitors in 2020 increased two‐fold compared to that in 2016, as there was evidence that these agents prevented the occurrence of cardiovascular events.^17^ Whereas the usage of dipeptidyl peptidase 4 (DPP4) inhibitors decreased, that of glucagon‐like peptide‐1 (GLP‐1) in 2020 analogs increased compared to that in 2016, partially because they both are incretins and cannot be administrated simultaneously to patients in Japan.^18^

We first observed the determinant for the current insulin therapy. Table 3 summarizes the baseline characteristics of the insulin‐untreated and ‐treated patients. Although there was no significant difference in the age of patients between the two groups, the duration of diabetes in the insulin‐treated patients was significantly longer than that in the insulin‐untreated patients (12.0 vs. 17.5 years, *p* = .0006). Furthermore, the HbA1c level was significantly higher in the insulin‐treated patients than in the insulin‐untreated patients (7.9% [62 mmol/mol] vs. 7.2% [55 mmol/mol], *p* < .0001). More important, both C‐CPI and UCPCR were significantly lower in the insulin‐treated patients than in the insulin‐untreated patients (0.9 vs. 2.2, *p* < .0001; 24.7 vs. 75.5, *p* = .0003, respectively; Table 3). The multivariate logistic regression analysis revealed that the diabetes duration in patients did not significantly differ between the groups. The HbA1c level, C‐CPI, and UCPCR exhibited a significant difference between the groups; the most significant difference was in C‐CPI (*p* = .0002). The cutoff of C‐CPI was 1.45 for the insulin‐treated group (area under the curve = 0.85241, sensitivity 85.0%, sensitivity 71.9%; data not shown).

**TABLE 3:** Analyses of the factors used for determining current insulin therapy. A total of 154 patients were examined to compare the patients who used insulin and those who did not in 2020. Group comparisons were performed as described in the Methods

Insulin therapy (2020)	No, median (range)	Yes, median (range)	Univariate *p*	Multivariate *p*
N	114	40	–	–
Sex (% male)	45.6	55.0	.3067	–
Age (years)	70.0 (43–89)	73.5 (33–89)	.7276	–
Duration of diabetes (years)	12.0 (6–28)	17.5 (7–32)	.0006*	.0594
Body mass index (kg/m^2^)	24.1 (15–36.7)	23.3 (15.4–2.9)	.1074*	.5365
HbA1c (%)	7.2 (5.5–10.5)	7.9 (6–14.8)	<.0001*	.0009*
eGFR (ml/min/1.73 m^2^)	67.0 (33.1–122.0)	72.2 (31.3–136.7)	.3828	–
UACR (mg/g Cr)	16.9 (1.7–1275.1)	23.4 (1.6–9722.4)	.0501*	.1600
C‐CPI	2.2 (0.5–8.8)	0.9 (0.2–3.57)	<.0001*	.0002*
UCPCR (mg/g Cr)	75.5 (9.3–465.6)	24.7 (2.5–191.0)	.0003*	.0301*

Abbreviations: C‐CPI, casual C‐peptide index; eGFR, estimated glomerular filtration rate; HbA1c, glycated hemoglobin; UACR, urine albumin creatine ratio; UCPCR, urine C‐peptide‐to‐creatinine ratio.

Next, we retrospectively examined the characteristics of patients who were introduced to insulin therapy after 4 years. Among the 154 patients, 116 who did not use insulin in 2016 were reevaluated, and 6 patients required insulin therapy. Table 4 presents the characteristics of the insulin‐unintroduced and insulin‐introduced patients; only the C‐CPI and the number of patients receiving GLP‐1 analogs significantly differed between the groups. Importantly, multivariate logistic regression analysis revealed that although treatment with both C‐CPI and GLP‐1 analogs contributed to the introduction of insulin, C‐CPI contributed the most (*p* = .0008; Table 4). The cutoff of C‐CPI was 1.45 for the patients to be introduced to insulin therapy (area under the curve = 0.82652, sensitivity 100.0%, sensitivity 63.6%; data not shown).

**TABLE 4:** Analyses of the factors affecting insulin introduction in the 4‐year period from 2016 and 2020. A total of 116 patients who had not used insulin in 2016 were examined to compare the patients who used insulin and those who did not in 2020. Group comparisons were performed as described in the Methods

Insulin introduction	No, median (range)	Yes, median (range)	Univariate *p*	Multivariate *p*
N	110	6	–	–
Sex (% male)	47.3	33.3	.5050	–
Age (years)	66 (39–85)	58 (29–75)	.0629*	.3540
Duration of diabetes (years)	8.5 (2–24)	12 (4–20)	.5649	–
Body mass index (kg/m^2^)	24.5 (16.6–37.4)	22.7 (19.6–30.8)	.8344	–
HbA1c (%)	6.9 (5.5–13.5)	7.5 (6.8–8.5)	.1405*	.3890
eGFR (ml/min/1.73 m^2^)	67.6 (37.8–106.7)	62.1 (52.8–132.2)	.4294	–
UACR (mg/g Cr)	11.0 (0–815.4)	11.3 (2.4–17.4)	.5192	–
C‐CPI	1.7 (0.3–4.4)	1.0 (0.4–1.5)	.0184*	.0008*
Sulfonylurea (%)	20.0	50.0	.0818*	.3206
Glinide (%)	17.3	16.7	.9695	–
Dipeptidyl peptidase‐4 inhibitor (%)	61.8	33.3	.1648	–
Biguanide (%)	43.5	50.0	.7555	–
α‐glucosidase inhibitor (%)	52.7	50.0	.8963	–
Sodium glucose cotransporter 2 inhibitor (%)	19.1	16.7	.8827	–
Thiazolidine (%)	3.6	0.0	.6345	–
Glucagon‐like peptide‐1 receptor agonist (%)	7.3	33.3	.0268*	.0056*

Abbreviations: C‐CPI, casual C‐peptide index; eGFR, estimated glomerular filtration rate; HbA1c, glycated hemoglobin; UACR, urine albumin creatine ratio.

Finally, we investigated the factors associated with the change in CPI (ΔCPI) for 4 years (Table 5) and found that the HbA1c level, C‐CPI, and usage of DPP4 inhibitors and glinide/biguanide positively correlated with ΔCPI. Interestingly, the multivariate logistic regression analysis revealed that the use of DPP4 inhibitors contributed the most to the unchanged CPI (Table 5).

**TABLE 5:** Analyses of the factors used for determining the difference in the casual C‐peptide index (C‐CPI) from 2016 to 2020. Group comparisons were performed as described in the Methods

ΔCPI (*N* = 154)	Univariate *p*	Multivariate *p*
Sex (% male)	.4469	–
Age (years)	.4481	–
Duration diabetes mellitus (years)	.5216	–
Body mass index (kg/m^2^)	.6680	–
HbA1c (%)	.0287*	.5514
eGFR (ml/min/1.73 m^2^)	.6833	–
UACR (mg/g Cr)	.6549	–
C‐CPI	.0007*	.0074*
Sulfonylurea (%)	.6072	–
Glinide (%)	.4916	–
Dipeptidyl peptidase‐4 inhibitor (%)	.0003*	.0010*
Biguanide (%)	.0335*	.8350
α‐glucosidase inhibitor (%)	.3778	–
Sodium glucose cotransporter 2 inhibitor (%)	.7126	–
Thiazolidine (%)	.2890	–
Glucagon‐like peptide‐1 receptor agonist (%)	.1014*	.0526
Insulin (%)	.5538	–

Abbreviations: C‐CPI, casual C‐peptide index; eGFR, estimated glomerular filtration rate; HbA1c, glycated hemoglobin; UACR, urine albumin creatine ratio.

## 4 DISCUSSION

Residual beta cell function is indispensable in patients with type 2 diabetes because it informs not only diagnoses but also the treatment itself.^16^, ^19^ Moreover, the function of pancreatic beta cells in patients with type 2 diabetes decreases gradually. However, patients with type 1 diabetes, who have a low endogenous insulin level, may experience more glucose fluctuation, thus making the patient more susceptible to reduced quality of life and complications of diabetes.^20^ Assessment of residual beta cell function at any time point would enable a more accurate assessment of pancreatic beta cell function, which will guide not only current therapeutic decisions but also future decisions.

To assess residual pancreatic beta cell function, we first examined factors contributing to current insulin therapy as they may be indirect markers for insulin deficiency owing to reduced beta cell function. HbA1c is a marker that indicates the requirement of insulin therapy, and typically, insulin therapy is initiated after considering the HbA1c level.^21^ We observed that although both indicators of insulin secretion, that is, C‐CPI, and UCPCR, influenced the requirement for insulin therapy, C‐CPI contributed the most (Table 3).

The C‐CPI was also useful in predicting the introduction of insulin therapy for 4 years (Table 4). Meanwhile, the UCPCR is not only a useful biomarker for insulin resistance but also applicable for differentiating type 2 diabetes from type 1 diabetes.^14^ Furthermore, we observed that the UCPCR was one of the useful markers for insulin therapy; however, the C‐CPI was more effective in predicting the need for insulin therapy. In contrast, some studies have demonstrated that the UCPCR is more useful than the C‐CPI. These contradictory findings may be because other studies analyzed blood or urine samples from fasting patients.^14^, ^15^, ^22^ Furthermore, in the current study, all test samples were collected at random time points depending on the availability of the patients. Considering that serum CPR and urine CPR are metabolized differently, the time of testing is a critical component for accurate assessment.^13^, ^23^ Our study indicated that at the time points when outpatients were tested, the C‐CPI was a more predictable marker than the UCPCR for insulin therapy.

Determining the changes in the C‐CPI might be necessary in the evaluation of other aspects in the future, as it can reveal the effect of current treatments on beta cell function. Here, we examined the factors associated with ΔC‐CPI over 4 years, demonstrating that the use of DPP4 inhibitors was most strongly correlated (except the initial C‐CPI). In fact, the findings of several basic science studies and clinical data have suggested that DPP4 inhibitors can preserve pancreatic beta cell function.^24^, ^25^, ^26^ Specifically, DPP4 inhibitors have been shown to mitigate endoplasmic reticulum stress, which often occurs in the beta cells of patients with diabetes, while also aiding in insulin production to compensate for this stress.^25^ Our results are consistent with this finding, and they indicate that ΔC‐CPI can serve as a critical biomarker for future therapy of type 2 diabetes.

When interpreting the current findings, several limitations have to be considered. We examined only “casual” tests for serum and urine, which may be affected by other conditions, such as the time of food intake before sample collection. In this respect, additional indices such as acute insulin response (AIR), AIRmax,^27^, ^28^ and glucagon testing^27^, ^29^ should be considered to evaluate residual insulin secretion. Additionally, this study involved a retrospective cross‐sectional design, had a small number of patients, and was performed in only a single hospital. Thus, the demographics of our patients may differ from those at other hospitals in Japan, particularly with respect to treatment. Although the present study included a longitudinal follow‐up of 4 years, a cause‐and‐effect relationship could not be discerned.

In conclusion, we reassessed the biomarkers to examine the function of pancreatic beta cells in type 2 diabetes and found that C‐CPI may serve as a convenient useful biomarker for not only the requirement current insulin treatment but also the requirement of insulin therapy for 4 years. Interestingly, we also observed that DPP4 inhibitors, which have been reported to preserve pancreatic beta cell function, were most strongly correlated with the changes in the C‐CPI, supporting the assumption that the C‐CPI can be used as a biomarker to examine the function of pancreatic beta cells. The C‐CPI could be determined easily and was found to be a more practical marker for outpatients; therefore, the present study results could have critical implications for primary care. Nevertheless, further studies are required with larger cohorts to confirm our conclusions regarding the strategy for insulin therapy.

## DISCLOSURE

The authors declare that they have no known competing financial interests or personal relationships that could have appeared to influence the work reported in this paper.
